# Futuristic Non-antibiotic Therapies to Combat Antibiotic Resistance: A Review

**DOI:** 10.3389/fmicb.2021.609459

**Published:** 2021-01-26

**Authors:** Manoj Kumar, Devojit Kumar Sarma, Swasti Shubham, Manoj Kumawat, Vinod Verma, Praveen Balabaskaran Nina, Devraj JP, Santosh Kumar, Birbal Singh, Rajnarayan R. Tiwari

**Affiliations:** ^1^ICMR-National Institute for Research in Environmental Health, Bhopal, India; ^2^Stem Cell Research Centre, Department of Hematology, SGPGIMS, Lucknow, India; ^3^Department of Epidemiology and Public Health, Central University of Tamil Nadu, Thiruvarur, India; ^4^ICMR- National Institute of Nutrition, Hyderabad, India; ^5^ICAR-Indian Veterinary Research Institute, Regional Station, Palampur, India

**Keywords:** antibiotic resistance, gene transfer, health problems, alternative therapies, drug-resistance

## Abstract

The looming problem of resistance to antibiotics in microorganisms is a global health concern. The drug-resistant microorganisms originating from anthropogenic sources and commercial livestock farming have posed serious environmental and health challenges. Antibiotic-resistant genes constituting the environmental “resistome” get transferred to human and veterinary pathogens. Hence, deciphering the origin, mechanism and extreme of transfer of these genetic factors into pathogens is extremely important to develop not only the therapeutic interventions to curtail the infections, but also the strategies to avert the menace of microbial drug-resistance. Clinicians, researchers and policymakers should jointly come up to develop the strategies to prevent superfluous exposure of pathogens to antibiotics in non-clinical settings. This article highlights the present scenario of increasing antimicrobial-resistance in pathogenic bacteria and the clinical importance of unconventional or non-antibiotic therapies to thwart the infectious pathogenic microorganisms.

## Highlights

- Drug-resistant Staphylococci, Enterococci and Streptococci are the major pathogens that increase morbidity, mortality and healthcare costs.
- Frenzied use of antibiotics as growth-promoters in some food animals is the prime driver of dissemination of antibiotic-resistant genes.
- Alternative therapies like stem cell-AMPs, CRISPR-Cas, probiotics, nanobiotics etc., should be explored to combat antibiotic resistant infections.

## Introduction

Discovery of antibiotics to treat infectious diseases had a phenomenal impact on human and animal health since 1940s. However, haphazard use of antibiotics and disinfectants has led to unprecedented health problems worldwide. This condition had its origin when microorganisms started developing genes that provide resistance toward residual antibiotics. The resistant genes augment the survival of pathogens in multiple environments, which not only limit the treatment options for infectious diseases, but also increase the morbidity and mortality by disseminating multiple drug resistant bacteria in human and animal. Antibiotic resistance, due to ESKAPE pathogens are found to be associated with high risk of mortality and morbidity leading to greater economic cost particularly in ICU settings of developing countries (Founou et al., 2017). The problem is aggravated further due to slow-pace inventions in the development of novel antibiotics (Nordmann et al., 2012; Kumar et al., 2016; Singh et al., 2017).

It is speculated that human deaths caused by drug-resistant microorganisms could rise from approximately 700,000 per year to 10 million per year by 2050 (World Health Organization, 2014). According to Laxminarayan et al. (2016), the world’s largest consumer of antibiotics have created conditions that promote drug-resistant infections. Poor public health conditions, lack of awareness about drug-resistant bacteria among the public, high incidence of diseases, easy availability of antibiotics and their haphazard use are the major factors aggravating the problem.

Vikesland et al. (2019) have summarized the mode of actions of some of the widely available antibiotics, which are the key drivers of development of antimicrobial resistance and subsequently transfer of resistant genes among pathogens; this condition is more prevalent in low- and high-income countries. Hence in order to have a stable and efficient drug-delivery system, use of CRISPR-Cas systems, nanotechnology-based approach – such as nanoparticles or nanocrystals as carriers of antibiotics, antibacterial and anti-viral chemical compounds (e.g., retinoid analogs), and synthetic and natural AMPs need to be considered (Lima et al., 2019; Pacios et al., 2020). Also for infections caused by multi-drug resistant pathogens, bacteriophages, mutant and bioengineered lytic phages and lytic endolysins alone or in combination with antibiotics need to be considered (Pacios et al., 2020).

Gupta et al. (2019) in their study have emphasized the potential of nanomaterials and nanoparticle-based approaches as self-therapeutic agents and drug-delivery vehicles as expected strategies against antimicrobial resistance.

In view of the dearth of developments in new antibiotics, there is an upsurge in use of computational and *in silico* tools to identify novel therapeutic targets (Sharma S. et al., 2019), and use of sustainable plant- and animal-origin and engineered products natural (Bérdy, 2012) and microorganisms (Kumar et al., 2016; Singh et al., 2016) as powerful supplementary therapeutics against infectious agents. Further advances in our understanding of properties and interactions among drug candidates, bacteria and metabolic pathways will pave the way to development of superior antimicrobial agents. Since combinatorial approaches have not proved to be vastly effective to develop potent drug molecules, development of antibiotics from already existing natural scaffolds could be an alternative short-term remedy against antibiotic resistance (Rossiter et al., 2017). Therefore, in the existing scenario, exploration and utilization of natural resources including probiotics and their metabolites will acquire eminence to deliver functional bio-molecules against MDR infections.

In the present review, we have highlighted the salient features of supplemental non-antibiotic therapies such as mesenchymal stem cell–derived AMPs, bacterial films and quorum-sensing inhibitors, immunotherapeutic, FMT and microbial or probiotic-based treatments.

## Drug Resistance as a Continued Process

Microorganisms are highly astute and evolve mechanisms swiftly to endure and proliferate in environments turning unfavorable. Although antibiotic-resistance started appearing soon after the clinical introduction of antibiotics, the problem was slow and ignored initially as a matter of low distress. Sulfonamide-resistant *Streptococcus pyogenes* appeared in the human clinical settings in early the 1930s, while penicillin-resistant *S. pyogenes* was noted in the 1940s. Emergence of multidrug-resistant bacteria was highlighted in the 1950s (Levy and Marshall, 2004; Table 1).

**TABLE 1:** Overview of the development of bacterial resistance against common antibiotics.

Antibiotic class	Mechanism of Action	Examples of antibiotics	Year introduced to the market	Year antibiotic resistance identified
Penicillins (β-Lactam)	Inhibition of bacterial cell wall synthesis.	Penicillin	1943	1940,1965, 1967,1976 (Ventola, 2015)
		Ampicillin	1961	1962,1964 (Fischer et al., 2010; Ravina, 2011)
		Amoxicillin	1972	1977 (Fischer et al., 2010; Roy, 2011)
		Methicillin	1960	1960 (Jevons, 1961)
Cephalosporins (β-Lactam)		Cefotaxime	1980	1983 (Knothe et al., 1983)
		Ceftaroline	2010	2011 (FDA, 2010; CDC, 2013)
	Avibactam inhibits serine β-lactamases enzyme	Ceftazidime (3^*rd*^ Generation cephalosporins)	1984	1987 (Burwen et al., 1994; Fischer et al., 2010; Humphries et al., 2015)
		Ceftazidime–avibactam	2015	2015 (FDA, 2015; Humphries et al., 2015)
Aminoglycosides	Protein biosynthesis inhibition	Streptomycin	1944	1946 (Youmans and Williston, 1946; Crofton and Mitchison, 1948; Schatz et al., 2005)
		Tobramycin	1967	1981 (Fischer et al., 2010)
		Amikacin	1976	1981 (Bongaerts and Kaptijn, 1981; John et al., 1982; Levine et al., 1985)
		Gentamicin	1963	1973 (Greene et al., 1973; Fischer et al., 2010)
		Neomycin	1952	1950 (Waisbren and Spink, 1950; Fischer et al., 2010)
		Kanamycin	1957	1967 (Umezawa et al., 1957)
Chloramphenicol		Chloramphenicol	1948	1960 (Smith and Worrel, 1949; Fischer et al., 2010; Lewis, 2013)
Glycopeptides	Cell wall synthesis inhibition	Vancomycin	1972	1988 (Anderson et al., 1961; Leclercq et al., 1988; Uttley, 1988; Murray, 2000; Depardieu et al., 2003; Sievert et al., 2008)
		Teicoplanin (derivative of Vancomycin)	1984	1986 (Johnson et al., 1990; Butler et al., 2014)
Ansamycins	RNA synthesis inhibition	Rifampin	1968	1972 (Tsukamura, 1972; Sensi, 1983; Telenti et al., 1993; Lesch, 2007)
Sulfonamides	DNA synthesis inhibition	Prontosil	1936, 1935	1942 (Lesch, 2007; Simon, 2011)
		Sulfamethoxazole	1961	1960 (Akiba et al., 1960; Stamm and Hooton, 1993; Andrew, 2013)
Tetracyclines	Protein biosynthesis inhibition	Tetracycline	1950	1959 (Ramsey and Edwards, 1961; Fischer et al., 2010)
Macrolide		Erythromycin	1953	1956 (Finland et al., 1956)
		Azithromycin	1980	2011 (Soge et al., 2012)
Oxazolidinones		Linezolid	2000	2001 (Tsiodras et al., 2001; Li and Corey, 2013; Török et al., 2016)
Quinolones	DNA synthesis inhibition	Ciprofloxacin	1987	2007 (Castanheira et al., 2007)
		Levofloxacin	1996	1996 (CDC, 2013)
Lipopeptides	Cell wall synthesis disruption disrupting multiple aspects of bacterial cell membrane function	Daptomycin	2003	2004 (Mangili et al., 2005)
		Bacitracin	1945	1955 (Weinberg, 1967; Mary, 2007)
		Aztreonam	1984, 1986	1986 (Res et al., 2017)
		Imipenem	1985	1996 (Troillet et al., 1997; Fischer et al., 2010)
Lincosamides	Interfering with the synthesis of proteins	Clindamycin	1966	1971 (Watanakunakorn, 1976; Troillet et al., 1997)

Two distinct pathways, namely vertical evolution (mutations which cause antibiotic tolerance transmittable to offspring), and horizontal evolution (acquisition of inheritable antibiotic-resistance genes from other bacteria via conjugation, transduction or transformation) are regarded as prime modes of development of antibiotic-resistance. The comprehensive genomic analysis of human and animal pathogens has shown that horizontal gene transfer is an important mechanism of transfer of antibiotic-resistant genes among microorganisms (Martínez et al., 2017).

The antibiotic resistance, though a grave concern, was overlooked for a long period (Martínez et al., 2017). However, incidences that attracted the attention of clinicians and biochemists included detection of bacteria carrying extended spectrum β-lactamases (ESBL) imparting resistance to penicillins and cephalosporins, extensively drug-resistant (XDR) *Mycobacterium tuberculosis*, and multidrug-resistant *Acinetobacter baumannii*, Enterobacteriaceae, *Neisseria gonorrhoeae*, and *Pseudomonas aeruginosa* (Wright, 2010a,b).

A decade ago, the New Delhi metallo-β-lactamase 1 (NDM-1) was first identified in a single isolate of *Klebsiella pneumoniae* and *Escherichia coli*, both isolated from a patient admitted in a hospital in New Delhi, India (Yong et al., 2009). Third-generation cephalosporin-resistant *E. coli* infections are accountable for the utmost disease burden and more than half of these infections occur in the community. This indicates that to reduce the burden of antimicrobial resistance (AMR), antimicrobial stewardship should be limited not only to the hospitals, but it is necessary to the primary care system including prescribers and interventions (OECD, 2019).

**FIGURE 1:**
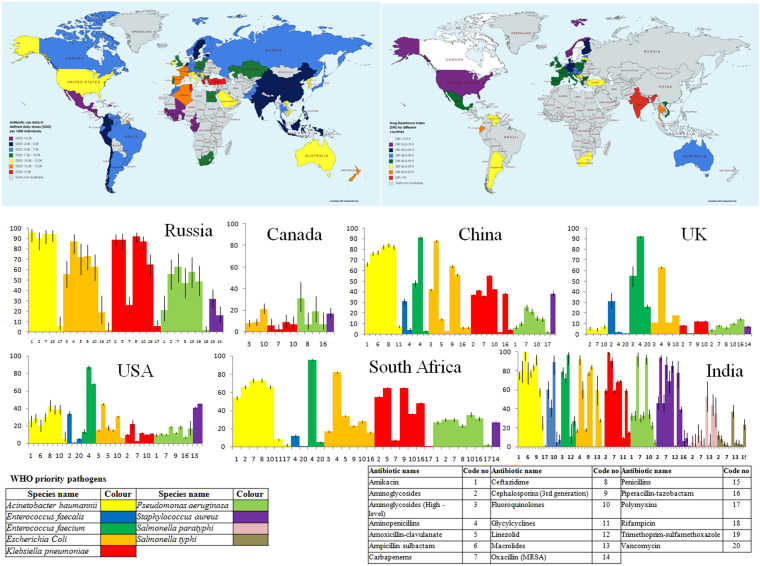
Country wide Antibiotic consumption rate (in defined daily doses (DD) per 1000 individuals) and Drug Resistance Index (DRI) along with prevalence of antibiotic resistance in some selected countries. [Data for antibiotic consumption rate (up to 2015) and prevalence of antibiotic resistance in the selected countries are retrieved from The Center for Disease Dynamics, Economics and Policy (The Center for Disease Dynamics, 2020). Drug Resistance Index map has been created from the data derived from Klein et al. (2019)].

Antibiotic-resistance is a globally admitted problem in clinical and health sectors, but more severe is in developing countries such as India (Ganguly et al., 2011). The crude mortality owing to infectious diseases in India was 416.75 per 100,000 persons, which was twice the rate prevailing in United States (roughly 200 per 100,000 persons; (Armstrong et al., 1999). It has been observed that there is a significant surge in the global consumption of antibiotics (increased by 65%, 21.1–34.8 billion DDDs (defined daily doses) during 2000 to 2015, which was mainly driven by low- and middle- income countries) (Klein et al., 2018). Recently, Klein et al., used an indicator (Drug Resistance Index, DRI) to elucidate the effectiveness of antibiotic therapy by combining antibiotic consumption and resistance (Klein et al., 2019). A highest DRI for low and middle- income countries was observed by using data from 41 countries on antibiotic uses and resistance in WHO priority pathogens. Figure 1 depicts the global consumption and DRI along with the drug resistance pattern in WHO priority pathogens against selected antibiotics in some countries.

**FIGURE 2:**
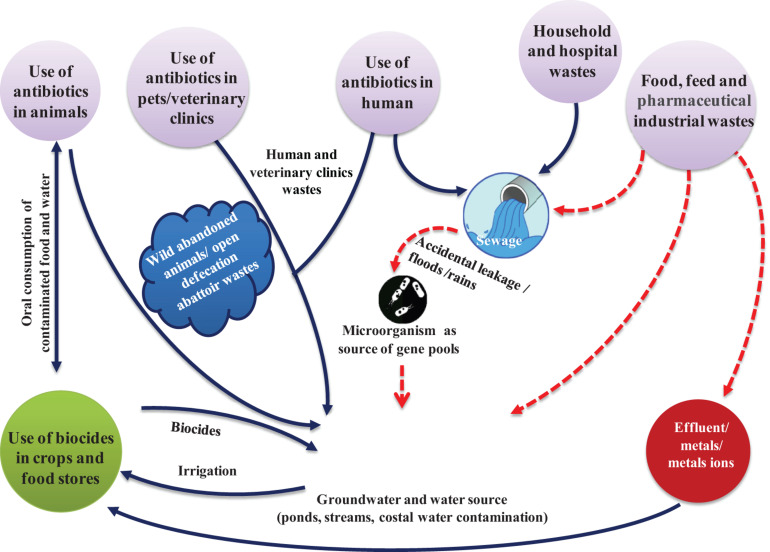
Factors leading to rapid development and use of antibiotics increasing antibiotic-resistance among pathogens, and the routes by which resistant pathogens enter human and animal food chain. Antibiotic-resistant bacteria from humans and animals enter water sources used for drinking or irrigation. Residual antibiotics released from industries alter the natural soil and water microbiota.

The surge in antibiotic-resistance might be due to poor public health systems, prevalence of infectious diseases and use of antibiotics without prescription of medical and veterinary specialists (Baquero et al., 2008). The threat is aggravated in poor or developing countries where medical services are scanty. Further, self-medication is a common practice as antibiotics are sold without restrictions in chemists’ shop, and the users are unaware of the consequences of overuse of antibiotics (Planta, 2007; Wellington et al., 2013; Figure 2).

## Antibiotics Usage and Its Consequence

The notion that antibiotics are safe to humans and veterinary health has changed. Besides killing microorganisms, the antibiotics interrupt human and animal health. Though antibiotics are recommended for human use after rigorous clinical trials of efficacy and safety, from a perspective of microorganisms, they pose health threats to humans. Medical professionals and regulatory agencies are dubious that some antibiotics over the long-term use could have debilitating health effects in humans (Merchant et al., 2018).

For instance, the fluoroquinolones, being safe to most people, are prescribed by medical doctors all over the world. The phenomenal side effects of antibiotics have urged health professionals and regulatory agencies to reassess their use in biomedical and veterinary segments. Side effects of fluoroquinolones, such as muscles and tendons damage, neuropsychiatric disorders and mitochondrial toxicity, are the compelling examples that antibiotics do to humans other than treating infections. Even though effective treatment of fluoroquinolone-associated disability (FQAD) is difficult, it is not so effective. Hence, in view of repeated incidences of FQAD, it is warned that the antibiotics should be prescribed exclusively for serious infections (Merchant et al., 2018).

## Livestock as a Major Contributor of Antibiotic-Resistance

Use of antimicrobials in animal feeding as growth-promoters is strongly correlated with emergence and spread of antimicrobial-resistant bacteria, making it difficult to treat infectious diseases. Many food-producing animals, especially the poultry, pigs and cattle are treated with antibiotics to prevent infections and to compensate for unhygienic conditions in commercial livestock farms. For instance, the antibiotic use in livestock in India is fourth highest in the world after China (23%), the United States (13%), and Brazil (9%) (Sivaraman, 2018). In United States, 80% of sold antibiotics are used in livestock alone (Mullard, 2015; Van Boeckel et al., 2015; FDA, 2017; Goutard et al., 2017). Nevertheless, non-therapeutic use of antibiotics in animal husbandry due to its contribution to increase antibiotic-resistant bacterial infections is of immense public health concern (Resistance, 2004).

The livestock act as a source that spreads resistant microorganisms into environment, for example, through contaminated bio wastes, milk or meat, or by direct contact with animals and humans working in animal farms. Similarly, the zoonotic pathogens act as donors of antimicrobial-resistant genes to human pathogens. As animals and livestock products are transported across the world, AMR distressing the food supply of one country poses threats in other places. For instance, after use of avoparcin, a glycopeptide antibiotic used as animal growth-promoter in Europe, vancomycin-resistant enterococci (VRE) spread in animal feed and meat (Wegener et al., 1999). Consequently, the avoparcin was banned in food-producing animals in European Union, and reduction has been noted in the incidences of VRE in humans and animals (Bates, 1997). Similarly, the spread of ciprofloxacin-resistant *Salmonella*, *Campylobacter* and *E. coli*, which have caused human infections difficult to treat, can be attributed to a significant extent to the use of fluoroquinolones (e.g., enrofloxacin) in milch and meat animals. There are several instances where drug-resistant bacteria spread worldwide via travel and food trade mode.

Bacteria develop resistance toward antibiotics when exposed to low doses over long periods. A common practice is to feed low-dose antibiotics to the livestock to promote body weight gain. In addition, antibiotics are also used arbitrarily to prevent diseases in crowded herds or flocks. Such practices contribute to emergence and spread of resistance to antibiotics. These practices lead to massive accumulation of antibiotics in the environment, and acquisition of resistance in microorganisms coming in contact with them (Allen, 2014). Many countries including European Union have imposed a ban on use of some antibiotics in feed or as growth promoters (Heuer et al., 2011).

In addition to the development of resistance to drugs due to mutations in genes, the microorganisms originating from non-clinical environments are the prime candidates expressing resistance toward antibiotics (Martinez et al., 2008). Therefore, attention is focused on understanding the sources and molecular mechanisms involved in acquisition of resistance.

However, it is difficult to determine the route and quantity of resistance factors from animals to humans and thus prove that animals act as reservoirs of resistant genes. This is further complicated by the spontaneously present resistance genes in the environment. For example, resistance to naturally occurring antibiotics is known to occur much before the progress of agriculture. The metallo-β-lactamases (of which NDM is one especially annoying example) have a very old origin, so ancient is the fact that no detectable sequence homology remains between different classes of these genes (Hall et al., 2004; Bebrone, 2007). Horizontal gene transfer is thought to play a significant role in the development of metallo-β-lactamases, but whether that process has been accelerated by using of antibiotics in agriculture is not known.

## Scale of Antibiotic Use in Human and Animals

As per 2010 statistical data, India with the consumption of 12.9 × 10^9^ units (10.7 units/person) was the largest consumer of antibiotics, followed by China, which used 10.0 × 10^9^ units (7.5 units per person), while the United States used 6.8 × 10^9^ units (22.0 units per person) (Van Boeckel et al., 2017). Seventy-six percent of the overall rise in antibiotic use during the decade 2000 to 2010 was noted in BRICS countries (Brazil, Russia, India, China, and South Africa) (Van Boeckel et al., 2015).

Among five major rising national economies i.e., BRICS countries, 23% of the rise in the retail antibiotic sales was attributed to India, while around 57% of the increase in medical sector was in China. Overall, in India, the pattern of antibiotic use is changing with decline in the use of ampicillin and co-trimoxazole and increase in quinolone consumption. The above scale-up in antibiotic use in India was due to swift economic growth, increasing incomes and incidences of infectious diseases (Laxminarayan and Chaudhury, 2016; Laxminarayan et al., 2016).

Several strategies are adopted to enhance animal production to maximize profits and fulfill the need of animal-origin foods. The global estimate of antimicrobial use in food-producing animals was at 63,151 (±1,560) tons in 2010, and was projected to rise by 67%, to 105,596 (±3,605) tons, by the year 2030. Out of this, 60% of the rise is estimated to be due to rising number of animals reared for food production. The remaining 34% rise was due to a shift in farming practices, with a larger proportion of animals projected to be the integral component of intensive farming systems by 2030. It is envisaged that, by 2030, the antimicrobial consumption in Asia could roughly reach 51,851 tons, representing 82% of the current global antimicrobial consumption in food animals in 2010. However, animal’s antimicrobial consumption is expected to grow by 99% by 2030 in the BRICS countries, whereas human antimicrobial consumption is expected to grow by 13% over the same period (Van Boeckel et al., 2015).

## Antibiotic-Resistant Genes (ARGs) as Environmental Contaminants

Antibiotics of human-origin enter the environment through a several routes. Antibiotics and their metabolites are released from hospitals through biological wastes (urine, feces, sputum, placenta, tissues and organs). Likewise, the antibiotics and antibiotic-resistant pathogens are released into environment through abandoned animals (e.g., cattle in India), stray animals (dogs, pigs, and birds) and open human defecation such as in slum area. From the wastewater treatment plants, the untreated antibiotics end up in sludge disseminated on fields as fertilizer or can be released as runoff directly into water (Hughes et al., 2012; Wellington et al., 2013). Sometimes, the wastewater is treated by releasing it into wetlands, thereby releasing antibiotics into water that directly or indirectly enter human and animals food chain (Scholz and Lee, 2005). A marked correlation has been noted between the routes of dispersal of antibiotics and resistant bacteria entering the human body (Baquero et al., 2008).

Impact of ARGs present in water on animal and human health needs further studies as does its use as a bacteriological indicator in terms of concentration and prevalence. Numerous decisive questions need to be answered in that aspect (Durso and Cook, 2018). There is a need to discover (1) whether the antibiotic resistance in real pathogens increases the risk of disease complications or results in increased cost of treatments of infections, and (2) whether presence of ARG in fecal indicators correlates in some way with high risk of horizontal gene transfer to pathogens still needs to be studied. At last, (3) the role of ARGs in environments in the emergence of antibiotic resistant pathogens needs to be assessed. There is a need to extensively study the interplay of environmental factors in relation to emergence of resistance which ultimately lead to evolve conceptual models for the role of environment in emergence and dissemination of resistance (Bengtsson-Palme et al., 2017).

People encounter with resistant microorganisms through various routes including drinking contaminated water, consuming contaminated vegetables, crops, fish, and meat. The bacteria once entered human body then transfer the ARGs to microflora inhabiting the host (Wellington et al., 2013).

## Gut Microbiota as a Reservoir for ARGs

Among various mechanisms facilitating the transfer of ARGs, the conjugative drug transfer of genetic determinants *viz*. plasmids and transposons, and transduction are important. However, the quantum and mechanism of ARG transfer from gut resistome to pathogens are still unknown. The macrolide-resistant genes *ermB*, *ermF*, and *ermG* and the tetracycline-resistant genes *tetM* and *tetQ* are likely to spread among other bacteria (Salyers et al., 2004). The ARGs from gut microorganisms tend to get transferred to related bacteria. For instance, the β-lactamase *cblA* present in Bacteroides is one of the most abundant ARGs in the microbiota of healthy persons as well as patients (Forslund et al., 2013). This gene hardly transfers to food-borne or opportunistic pathogens, such as Enterobacteriaceae, even though functional metagenomic analysis indicates that this gene β-lactamase is resistant to human *E. coli* (Sommer et al., 2009; Buelow et al., 2014).

It has been noted that transfer of resistant genes between unrelated bacteria, for example between anaerobic gut commensals (e.g., Bacteroides) and Gram-negative facultative anaerobic opportunistic pathogens (e.g., *Enterobacter*) does not occur readily. Even then conjugative transfer of ARGs from Bacteroides to *E. coli* is possible under controlled laboratory environment (Guiney and Davis, 1978; Privitera et al., 1979), however, within the gut, the conditions are adverse for such gene transfer.

With the use of metagenomics as a tool, it is possible to detect pathogens in the fecal samples (Loman et al., 2013), and track fluctuations in individual patients (Buelow et al., 2014). Probably, the diagnostic relevance of resistome profiling by metagenomic analysis is limited, performing sequencing and analyzing the metagenomic data are time-consuming processes (Singh et al., 2008). The advancement in high-throughput genomic sequencing technologies enabled rapid and reliable mapping of resistome in gut microbiome. This will guide the choice of antibiotics for curtailing infections, at least partially, by the composition and relative abundance of the ARGs in gene reservoir in patients. To fully assess the risks that are associated with the selection of ARGs in GI or genitourinary commensals, profiling of their ARGs and study of horizontal gene transfer is required.

The normal human microbiota is one of the main reservoirs of ARGs that can be transferred to pathogenic bacteria, which come in contact during disease progression. Dissemination of resistant microorganisms and ARGs occurs between humans during direct contact and spread into human-associated environments.

Studies have shown that multidrug-resistant uropathogenic *E. coli* (UPEC) share the same genetic lineages with the *E. coli* that infects poultry; this suggests that there is a high likelihood of food-borne transmissions of antibiotic-resistant UPEC and its genetic matter. To accurately quantify the contribution of food or food animals, to evaluate the impact of environmental samples as reservoirs for antibiotic-resistant bacteria and to plot their route of transmission to humans, modern technological tools such as whole genome sequencing (WGS) and metagenomics should be used. Such approaches will help develop focused public health interventions to prevent the infections.

## Alternative Non-Antibiotic Strategies to Combat Antibiotic-Resistant Pathogens

**FIGURE 3:**
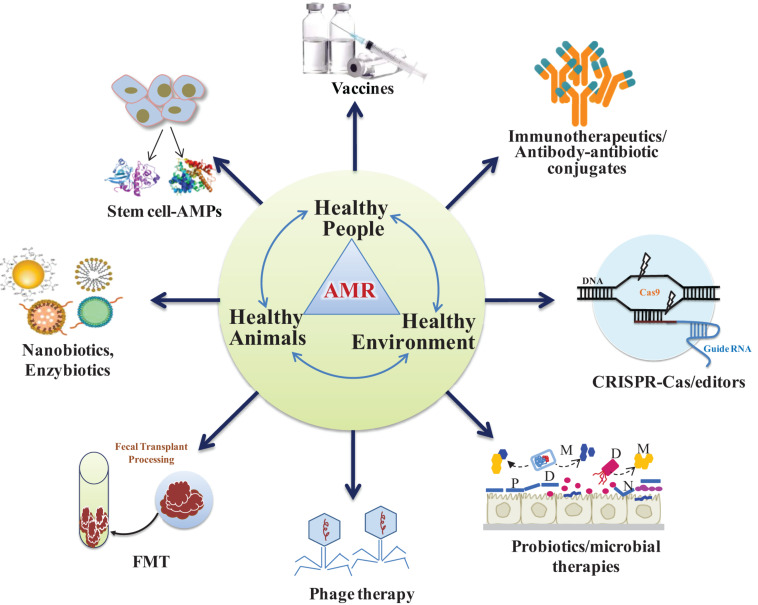
Alternative non-antibiotic strategies to prevent antimicrobial-resistant infections in animals and humans. Probiotics and phages act as microbiome-modifying therapies to defend against drug-resistant pathogens. Healthy people, healthy animals and healthy environment constitute as One Health Concept to keep environment healthy (P, prebiotics; D, designer or engineered probiotics; M, microbial metabolites; FMT, fecal microbiota transplantation).

It is imperative to evolve alternative non-antibiotic strategies that are safer to humans and livestock and effective against infectious pathogens (Singh et al., 2014; Kumar et al., 2016). Use of bacteriophage (Kadouri et al., 2013; Shatzkes et al., 2017a,b), antimicrobial peptides (AMPs) or bacteriocins (Cotter et al., 2013; Kumar et al., 2016; Garcia-Gutierrez et al., 2019), antimicrobial adjuvants, fecal microbiota transplant (FMT) and competitive exclusion of pathogens through genetically modified probiotics and postbiotics are the prospective alternative unconventional strategies (Figure 3). In this context, research should also be carried out in finding ideal targets for new inhibitory molecules like bacterial secretion system and two component system. Bacterial secretion system is a highly specialized nano-mechanical system analogous to “nano-syringes” that are capable of direct delivery of substances in eukaryotic cells. This makes it a very desirable tool for nano-therapeutics and targeted drug delivery system. Of the six families known (type I–VI secretion systems), only the type III, IV, and VI systems have been shown to facilitate direct delivery into the cytoplasm of a target cell (Walker et al., 2017). Such targeted delivery of antimicrobials may reduce the rapidity of antimicrobial resistance evolution. In addition, Bacterial type III secretion system (T3SS) is an attractive target for developing antibacterial as it is essential in the pathogenesis of many Gram-negative bacteria (Mcshan and De Guzman, 2015). On the other hand, two component system (TCS) are global regulatory elements unique to bacteria that are essential for growth and virulence. The fact that they are not present in eukaryotic cells makes them a potentially attractive target for future antimicrobials. Several TCS involved in cell cycle and cell envelope integrity have been identified and show promise as targets for novel antimicrobials in a bid to alleviate MDR (Cardona et al., 2018).

## Stem Cell-Derived Antimicrobial Peptides

Mesenchymal stem cells (MSCs) have been extensively studied for several decades to develop a safe and promising therapeutic product against a broad range of chronic diseases. MSCs exhibit promising ability to promote immunomodulation, tissue healing and control of excessive inflammation (Harman et al., 2017). Recently, it was established that human MSCs synthesize factors that behave as antimicrobial peptides (AMPs) that eradicate the bacteria through multiple mechanisms including inhibition of bacterial cell wall synthesis (Alcayaga-Miranda et al., 2017; Cortes-Araya et al., 2018; Marx et al., 2020). Therefore, secretome from MSCs, which significantly reduces the bacterial infections including the antibiotic-resistant MRSA, represents a hopeful approach or supportive treatment in future against various related infections.

Conditioned medium containing AMPs named as lipocal, hepcidin and LL-37 derived from human bone marrow (BM) and umbilical-cord MSCs (hUCMSCs) was found to exhibit antibacterial effects against drug resistant clinical pathogens such as *E. coli*, *S. aureus* and *K. pneumonia* (Mccarthy et al., 2020). hUMSCs possessed direct antagonistic activities against imipenem-resistant *P. aeruginosa* isolated from human infants (Ren et al., 2020).

## Hemofiltration Devices

Suppressing the cytokine storm is important to prevent organ damage in several cases of critical illnesses and infections. Hemofiltration or renal replacement therapies used in intensive clinical care settings utilize the devices that bind to and remove circulating bacterial products, inflammatory mediators and cytokines (Liu et al., 2018) and some pathogens circulating in blood. Such devices, for example, extracorporeal pathogen removal filters are at various stages of development. Two of the more fascinating devices include the mannose-binding lectins (Mccrea et al., 2014) or bound heparin (Kang et al., 2014). It is thought that if substantial reduction in the pathogenic bacterial load is achieved by these hemofilters, the host immune system will be able to tackle the remaining pathogens even in cases of multidrug resistance.

Hemofiltration is useful in curtail the adverse effects of infections in infants and aged patients. It was found to reduce the serum levels of bile acid microbial metabolites namely, total bilirubin, direct bilirubin, total bile acids, lactate and IL-6 in patients suffering from bacterial sepsis and hepatic dysfunction (Cui et al., 2018). Various devises are available in markets for hemofiltration.

## Quorum-Sensing Inhibitors

Formation of biofilms and quorum sensing are the two important attributes of bacterial species enhancing the chances of their survival under adverse environments. Quorum sensing is one of the main methods of intracellular communication between bacteria. An array of natural and synthetic molecules can block quorum sensing, which are under study in experimental models with interesting results. Several classes of compounds with potential quorum-sensing inhibitions are reported (Saeki et al., 2020). Whether quorum sensing inhibitors could ever be of practical clinical significance in preventing infections is a subject of considerable ponders (Kang et al., 2014; Brackman and Coenye, 2015).

## CRISPR-Cas Against Antimicrobial Resistant Pathogens

Clustered regularly interspaced short palindromic repeats (CRISPR)-cas is a distinctive adaptive immune feature in archaea and bacteria that provides protection against invading bacteriophages (Barrangou et al., 2007). Short sequences from bacteriophages or plasmids called as spacers are inserted into the bacterial genome as CRISPR array; the guide RNAs from the spacers will be utilized by the Cas protein machinery for specific targeting of the invading nucleic acid carrying the same sequence (Pursey et al., 2018; Shabbir et al., 2019). CRISPR-Cas systems are classified into two classes: class I comprises of types I, III, and IV, while class II consists of types II, V, and VI (Pursey et al., 2018). In Class I, multiple Cas proteins participate in DNA recognition and cleavage, whereas in class II, a single multi-domain Cas protein recognizes and cleaves the DNA (Pursey et al., 2018). The later (type II CRISPR-Cas9 system) has revolutionized molecular biology in the last decade and is used in genome editing of prokaryotes and eukaryotes. Several groups have shown the use of CRISPR-Cas9 in selective removal of AMR genes from bacterial populations (Bikard et al., 2014; Citorik et al., 2014). Phagemids and conjugative plasmids have been shown to deliver CRISPR-Cas for selective targeting of AMR genes in plasmids and chromosome, respectively (Bikard et al., 2014). Removal of AMR genes results in the sensitization of the bacteria to antibiotics (Citorik et al., 2014). CRISPR-Cas9 phagemids can kill specific bacteria *in vivo* (Bikard et al., 2014; Citorik et al., 2014). Delivering CRISPR-Cas through temperate phages can provide selective advantage to re-sensitized bacteria (Yosef et al., 2015). A synthetic biology strategy that utilizes engineering phage genomes in *Saccharomyces cerevisiae* has been shown to modulate the phage host spectrum (Ando et al., 2015). To circumvent the loading and packing efficiency in phage-based delivery, a successful non-viral editing strategy involving nano-sized CRISPR complexes (polymer-derivatized Cas9 complexed with single guide RNA) has been developed. Nanosized CRISPR complexes can effectively target the mec-A gene involved in methicillin-resistance inside the methicillin-resistant *S. aureus* (Kang et al., 2017). Despite the promise, there are some major challenges in the use of CRISPR-Cas9 system against AMR genes. These include perturbations in the microbial community after the removal of AMR bacteria, narrow host range of CRISPR-Cas vectors, resistance due to anti-CRISPR genes, and legislation (Pursey et al., 2018).

## Development and Use of Immunotherapeutic and Vaccines

As health authorities, clinicians and drug developers struggle with the emerging antimicrobial resistance (AMR) crisis, vaccines are considered as a potential solution. Immunotherapeutic are the biomolecules that boost the immune system of host and confer immunity against infectious agents. One of the most widely used immunotherapeutic agents is pegfilgrastim, a granulocyte colony-stimulating factor (G-CSF). It is used to increase the severely decreased neutrophil count in patients undergoing chemotherapy (Molineux, 2004).

Notably, it is important to maintain an appropriate neutrophil count in the blood so that the immune system can combat infections. Similar therapy is also used in animals in the form of pegbovigrastim, a bovine G-CSF used in cattle before parturition to improve immunity and reduce the incidences of mastitis. The advantage of immunotherapeutic agents lies in the fact that they boost the internal immune system. However, the disadvantage is the precise determination of timing of delivery.

Developments in new recombinant vaccine technology have been instrumental in reducing primary and secondary bacterial infections that would have necessitated the use of various antibiotics. Vaccines continue to be one of the most significant ways to prevent infections. A few of vaccines against deadly pathogenic bacteria, for example, *Clostridium difficile* (Phase III), *Mycobacterium tuberculosis* (Phase II), Group B *Streptococcus* (Phase II), *S. aureus* (Phase II) are in mid-stage clinical development by pharma companies.

## Phage Therapies

Failure of currently available antibiotics to treat some infections is worrying biomedical problem (Wright et al., 2019; Pacios et al., 2020). Phage therapy is extensively investigated as an alternative therapy to combat bacterial infections. This therapy was introduced in the early 1920s in Georgia and later on in whole Eastern Europe and also western countries (Wittebole et al., 2014). Even though there are several challenges, the bacteriophage therapy has potential to be used as a substitute for antimicrobial agents against drug-resistant pathogens in future. The technique is gaining popularity in present scenario because phages are ubiquitous, host-specific and harmless and can be administered orally along with food (Wittebole et al., 2014).

Recombinant phages are developed to deliver antimicrobial proteins in target bacteria. The therapy can be used topically on open wounds (Wright et al., 2009), or given intravenously in case of systemic infections. However, there are some serious concerns associated with phage therapy. The major one is their fine specificity toward host bacterium species. This precludes their applications as empiric therapy for acute infections.

Further, it is essential that pathogen must be identified before selecting a phage as therapeutic strategy. Developing and establishing a complete library of phages for every plausible infectious bacteria will be a big challenge (Wittebole et al., 2014). In addition, the therapy requires knowledge of the target sites. As it is likely that bacteria might develop resistance to phages, the phage libraries should be continuously screened for their efficacy to curtail bacterial pathogens.

Bacteriophage lysins, the extremely specific peptidoglycan hydrolases, were the base for their investigation as antibacterial agents and they were also named “enzybiotics” (Nelson et al., 2001). Owing to their modular structure, synthesis and use of bioengineered lysins with designed properties such as higher lytic activities or broader spectrum bacteriophages lysins are promising prospects. Lysins can be engineered to kill several pathogens including Gram-negative bacteria. These enzymes have attractive features that they do not activate an adverse immune response and raise of resistance is very unlikely.

As multidrug-resistant pathogens become a greater and more widespread threat, engineered lysins represent a newer modality of therapy, which is powerful and readily available to fight antimicrobial resistance (Vazquez et al., 2018).

## Fecal Microbiota Transplant (FMT)

Fecal microbiota transplant therapy is known by a variety of names as fecal bacteriotherapy, fecal transfusion, fecal transplant, fecal enema, human probiotic infusion, and stool transplant. But, in veterinary medicine, it is known as “transformation” used for treatment of ruminant animals such as sheep, cow, etc. (Depeters and George, 2014). The introduction of FMT into mainstream medicine was first described in 1958 to treat four ill patients suffering from pseudomembranous colitis (Eiseman et al., 1956). However, the first use of fecal enema therapy was described by Ge Hong in fourth-century China (Zhang et al., 2012). FMT is the process of transplantation of a suspension of fecal matter containing commensal bacteria from a healthy individual donor using various routes including enema, nasogastric, nasoduodenal and colonoscopy into intestinal lumen of the recipients (Bakken et al., 2011; Smits et al., 2013). Few studies have shown that FMT is an effective treatment regimen for people with *C. difficile* infection along with other gastrointestinal diseases, such as irritable bowel syndrome, colitis, constipation, diarrhea and several other neurological conditions such as Parkinson’s, multiple sclerosis, etc. However, the safety and efficacy of FMT therapy is related to ethical issues; therefore, appropriate clinical trials, data and deep scientific research are needed for its approval as therapy owning to its new hope to save the humanity from antibiotic resistance menace.

Clinical trials have revealed that autologous FMT (aFMT) is better than probiotic therapy and induced a speedy and almost complete recovery of GI microbiota in antibiotics-perturbed human patients (Suez et al., 2018). Intention-to-treat clinical trial involving 22 patients in donor FMT group revealed that 20 of 22 (90.9%) patients achieved clinical cure from *Cl. difficile* infection. The success rate of clinical recovery was higher than aFMT (62.5%), and donor FMT restored the gut microbial diversity and functioning of the recipients comparable to the donors (Kelly et al., 2016). High-intensity FMT treatment in adult patients with mild to moderate ulcerative colitis (UC) resulted in high likelihood of remission at 8 weeks of the treatment. Though further research is suggested, it was observed that FMT had better outcomes than the aFMT (Costello et al., 2019).

## Nanoantibiotics

Nanoparticulate materials can either be used to deliver antimicrobial substances or may contain antimicrobial substances. The metal and metal oxide-based nanoparticles and antibiotics, due to less toxicity and enhanced antibacterial, antiviral and anticancer efficacy, are regarded as promising therapeutic candidates for future applications in biomedical sciences (Fernandez-Moure et al., 2017; Muzammil et al., 2018). Their size provides them with unique properties such as an increased surface area to volume ratio, which makes them efficient drug carriers and enhance their solubility, compatibility as well as ease of delivery (Wang et al., 2017). Nanoparticles, in addition to acting as carriers for targeted drug delivery, can have antibacterial properties of their own via several mechanisms such as disruption of bacterial wall, biofilm inhibition, modulation of immune response in host, generation of reactive oxygen species and damage to key DNA and protein molecules of the resistant bacteria (Baptista et al., 2018). Due to these diverse mechanisms of action, nano-antibiotics are likely to be effective against antibiotic resistant bacteria. Recently researchers demonstrated that bismuth nanoparticles exhibit broad anticandidal activity and slow down the spread of the multidrug-resistant *Candida auris* strains in the healthcare-settings (Vazquez-Munoz et al., 2020). However, detailed study of pharmacokinetics, precision of action and controllability of the nanoantibiotics need to be conducted to ensure its efficacy and safety in clinical settings.

## Probiotics, Postbiotics and Synbiotics

Identification of novel animal-origin probiotics, and postbiotics, the non-viable microbial probiotics or probiotic metabolites that have biological activities in host (Tsilingiri et al., 2012; Aguilar-Toalá et al., 2018), and using them as alternative therapeutic combinations may facilitate the development of improved dosing regimens and strategies to prevent economic loss due to enteric infections.

Probiotics, the live microorganisms or microbial feed supplements primarily comprise of two classes of lactic acid-producing microorganisms: the Bifidobacteria and lactic acid bacteria (LAB) including species of *Enterococcus*, *Lactobacillus*, *Lactococcus*, *Pediococcus*, *Vagococcus*, *Aerococcus*, *Carnobacterium*, *Streptococcus*, and *Weissella.* Most LAB, since generally regarded as safe status, and abundance of some genera in GI tract, mammary gland and feminine genitourinary tract (Singh and Madhup, 2013) are regarded as alternative health-promoting strategies. Advances in next generation sequencing and genetic engineering has enabled scientists to develop future strategies, such as bioengineered probiotics or pharmabiotics, which may become a bio-therapeutic or prophylactic strategy against bacterial infection. Bioengineered probiotics with manifold immunogenic properties could be a possible option against antibiotics. Engineered or recombinant probiotics could be personalized to deliver drugs, therapeutic proteins and gene therapy vectors with great competence, with a higher degree of site specificity than common drug administration regimes.

Vaccinations using recombinant probiotics against *Yersinia pseudotuberculosis*, *Salmonella enterica*, enterotoxigenic *E. coli*, and *Streptococcus pneumonia* (Wu and Chung, 2007; Daniel et al., 2009; Hernani et al., 2011; Kajikawa, 2012) have generated desirable immune responses in murine models. Recombinant probiotic bacteria *Lactobacillus acidophilus* and *Lactobacillus gasseri* were used to deliver protective antigen and exhibit anti-protective antigen antibody and T-cell–mediated responses against *Bacillus anthracis* (Mohamadzadeh et al., 2009). These promising therapies deserve further assessment before they are recommended for human use.

## One Health Model to Tackle AMR

Prevention of AMR is associated with the One Health concept. Since antibiotic resistance genes are persistent in environmental and human–animal health interfaces, an approach which deals with all the three areas is required, which highlights the concept of “One Health approach” (Chee-Sanford et al., 2009). The One Health approach is defined as “the mutual attempt of various disciplines- working locally nationally, and worldwide – to accomplish optimal health for humans, livestock and our environment” (Fletcher, 2015). This approach perceives that human health is linked to the health of animals and the environment and is applicable to the crisis of antibiotic resistance as well. Dissemination of ARGs through livestock is due to use of many antibiotics in animal rearing, in sub-therapeutic doses and with elongated exposure periods, these production systems generate perfect milieu for bacteria for horizontal gene transfer that confers resistance. ARGs can consequently be transmitted to human-adapted pathogens or other gut microorganisms. Since the antibiotics used for both animals and human beings are similar or closely related, as are most of the animal and human pathogenic bacteria, similar patterns of antimicrobial-resistance are likely to emerge, in addition transmission of such resistance through genetic transfer between animal and human pathogens is highly possible, either directly or via the environment.

The One Health approach for dealing with AMR, encircling all three pillars (human health, animal health, and environmental health), will depend on sound knowledge about the interactions and the ways how these components interrelate in the transmission to humans.

## Outlook and Challenges

While pressure is to increase production from the livestock sector, maintaining human and animal health is also essential. Even though the overall burden of drug-resistance is difficult to estimate, it seems reasonable to believe that the burden will be high in vulnerable groups such as immune-compromised elderly persons, patients and the neonates.

Indeed, antibiotics therapies have transformed treatments against bacterial and fungal infections. However, widespread infections are posing threats to human and animal health with several pathogens developing resistant to available antibiotics. The normal role of antibiotics in social context of the microbial communities is not clear. While some examples such as ARGs dissemination between environmental and pathogenic bacteria are evident, the intricacy of the mechanisms and relative paucity of the observations made so far are lacking. Processes and relative paucity of studies indicate that knowledge is still missing in the field.

Novel branded antibiotics, namely besifloxacin, ceftobiprole, ceftaroline, dalbavancin, delafloxacin, omadacycline, ozenoxacin, oritavancin, telavancin, and tedizolid are effective against drug-resistant Gram-positive infectious bacteria (Koulenti et al., 2019). However, it is likely that pathogens may develop resistance against these antibiotics as well. It is, therefore, imperative to use combinations of non-antibiotic therapies against infectious pathogens.

Developing alternative methods of producing animal-origin foods is one way to minimize environmental pollution and development of superbugs and AMR genes (Singh, 2020). We strongly posit that there is need to screen and utilize natural plant (Wright, 2017) and animal products (Singh et al., 2019) as alternative therapeutics. In addition, cutting edge molecular approaches, nanotechnology-oriented methods (Vikesland et al., 2019), genome and proteome databases of stem cells and microorganisms (Sharma R. et al., 2019) to identify potential bio-molecules as futuristic antimicrobial candidates against drug-resistant pathogens. Bioengineered microorganism or probiotics as drug-delivery vehicles, and their metabolites (Kumar et al., 2016) is another viable option to curb drug-resistant pathogens.

In conclusion, antibiotic resistance can affect the people or animals at any stage of life. It is advisable to develop alternative therapies to lessen the dependence on chemical therapeutics. The efficacy of antibiotics is waning since they became a part of modern medicine before seven decades. Experts from diverse fields such as clinical research, microbiology, genetics and computational engineering, imaging and modeling should work in combination to evolve strategies and develop novel therapeutics to tackle the problem. Clinicians should avoid unnecessary prescription and over prescription of antibiotics to the patients having normal infections and advise the patients to follow good hygiene such as hand washing and appropriate infection control measures.

## Author Contributions

All authors listed have made a substantial, direct and intellectual contribution to the work, and approved it for publication.

## Conflict of Interest

The authors declare that the research was conducted in the absence of any commercial or financial relationships that could be construed as a potential conflict of interest.
